# Risk-prediction models for intravenous immunoglobulin resistance in Kawasaki disease: Risk-of-Bias Assessment using PROBAST

**DOI:** 10.1038/s41390-023-02558-6

**Published:** 2023-03-24

**Authors:** Shuhui Wang, Hongbiao Huang, Miao Hou, Qiuqin Xu, Weiguo Qian, Yunjia Tang, Xuan Li, Guanghui Qian, Jin Ma, Yiming Zheng, Yueping Shen, Haitao Lv

**Affiliations:** 1 Department of Cardiology, Children’s Hospital of Soochow University, Suzhou, Jiangsu 215003 China; 2 Department of Pediatrics, Institute of Pediatric Research, Children’s Hospital of Soochow University, Suzhou, Jiangsu 215003 China; 3 Department of Pharmacy, Children’s Hospital of Soochow University, Suzhou, Jiangsu 215003 China; 4 Department of Epidemiology and Biostatistics, School of Public Health, Medical College of Soochow University, Suzhou, Jiangsu 215123 China

## Abstract

**Background:**

The prediction model of intravenous immunoglobulin (IVIG) resistance in Kawasaki disease can calculate the probability of IVIG resistance and provide a basis for clinical decision-making. We aim to assess the quality of these models developed in the children with Kawasaki disease.

**Methods:**

Studies of prediction models for IVIG-resistant Kawasaki disease were identified through searches in the PubMed, Web of Science, and Embase databases. Two investigators independently performed literature screening, data extraction, quality evaluation, and discrepancies were settled by a statistician. The checklist for critical appraisal and data extraction for systematic reviews of prediction modeling studies (CHARMS) was used for data extraction, and the prediction models were evaluated using the Prediction Model Risk of Bias Assessment Tool (PROBAST).

**Results:**

Seventeen studies meeting the selection criteria were included in the qualitative analysis. The top three predictors were neutrophil measurements (peripheral neutrophil count and neutrophil %), serum albumin level, and C-reactive protein (CRP) level. The reported area under the curve (AUC) values for the developed models ranged from 0.672 (95% confidence interval [CI]: 0.631–0.712) to 0.891 (95% CI: 0.837–0.945); The studies showed a high risk of bias (ROB) for modeling techniques, yielding a high overall ROB.

**Conclusion:**

IVIG resistance models for Kawasaki disease showed high ROB. An emphasis on improving their quality can provide high-quality evidence for clinical practice.

**Impact statement:**

- This study systematically evaluated the risk of bias (ROB) of existing prediction models for intravenous immunoglobulin (IVIG) resistance in Kawasaki disease to provide guidance for future model development meeting clinical expectations.
- This is the first study to systematically evaluate the ROB of IVIG resistance in Kawasaki disease by using PROBAST. ROB may reduce model performance in different populations.
- Future prediction models should account for this problem, and PROBAST can help improve the methodological quality and applicability of prediction model development.

## Introduction

Kawasaki disease, also known as cutaneous mucosal lymph node syndrome, is an acute febrile disease presenting with systemic vasculitis as the main lesion. The incidence of Kawasaki disease is increasing, and it has become the main cause of acquired heart disease in most developed countries and regions.^1–3^ Coronary artery lesions (CALs) are the most common and serious complication of Kawasaki disease, and these lesions can lead to long-term sequelae such as coronary stenosis or obstruction.

The American Heart Association (AHA) and American Academy of Pediatrics (AAP) advise high-dose IVIG (2 g/kg) combined with acetylsalicylic acid (ASA) as the first-line therapy for KD, which can also reduce the risk of CALs.^4^ However, 15–20% of children with KD are insensitive to initial IVIG treatment, presenting with persistent or recurrent fever and showing a greater risk of developing CALs.^5,6^ Severe complications such as Kawasaki disease shock syndrome (KDSS) or Kawasaki disease complicated by macrophage activation syndrome (KD-MAS) may also occur, endangering the lives of children.^7,8^ Thus, early prediction of the disease course and additional effective treatment for children with Kawasaki disease to prevent the occurrence of CALs may be important.

In the past few years, researchers in several countries and regions have analyzed the clinical data of children with Kawasaki disease and developed predictive scoring models for IVIG resistance based on clinical signs, symptoms, and laboratory tests. However, these prediction models have shown difficulty in meeting clinical expectations, and there is no consensus on systematically synthesizing these prediction models. Moreover, the prediction models themselves did not work well in different populations or the studies did not perform external population validation.^9–13^

The Prediction Model Risk of Bias Assessment Tool (PROBAST) is useful for assessing prediction model studies and critically appraising prediction model studies.^14,15^ It includes 20 signal questions across four domains (Participants, Predictors, Outcomes, and Analysis) that can be used to evaluate the risk of bias (ROB) of the prediction models. An ROB assessment of IVIG resistance prediction models has not been reported to date. We used PROBAST to provide a standard method for evaluating IVIG-resistant Kawasaki disease prediction models, which can help evaluators evaluate model quality in a structured and transparent manner. Thus, the purpose of this study was to identify and evaluate the existing prediction models for IVIG resistance risk that have been developed or applied to the Kawasaki disease population. We used PROBAST to evaluate the ROB in studies reporting Kawasaki disease IVIG resistance prediction models and thereby aimed to provide an objective basis for clinical application as well as guidance for future model development and updates.

## Methods

We designed this study in accordance with the checklist for critical appraisal and data extraction for systematic reviews of prediction modeling studies (CHARMS)^16^ and used the PROBAST to assess the ROB of the studies. We presented this study in accordance with the Preferred Reporting Items for Systematic Reviews and Meta-Analyses (PRISMA). This protocol was registered on PROSPERO (Prospero registration number: CRD42022312740), We used published articles from open access databases, so specific patient consent and ethics committee’s approval were not required.

### Literature search

We systematically searched PubMed and Embase databases as well as the Web of Science for English-language studies published from June 2006 to October 2021 and reporting a prediction model for IVIG-resistant Kawasaki disease. Searches were performed using the following search algorithm: ((Kawasaki disease) OR (mucocutaneous lymph node syndrome)) AND ((IVIG resistance) OR (IVIG unresponsiveness)) AND ((predict) OR (score) OR (nomogram) OR (model)). The literature search details of the strategy are presented in Supplementary Table 1. Two researchers (WS, HH) conducted the literature search independently, and the differences between the findings obtained by the two researchers were reviewed and resolved by a third researcher (SY). To find other eligible studies, we also performed manual searches of the reference lists of each eligible article.

### Eligibility criteria

We included all reported model development studies on IVIG-resistant Kawasaki disease. Table 1 shows a detailed description of the PICOTS for the study. The inclusion criteria were as follows: (1) predictive models established in the Kawasaki population and meeting the Japanese Kawasaki disease diagnostic criteria or the AHA common standards; (2) the prediction model included at least two predictors because the purpose of PROBAST is to assess multivariate predictive models for diagnosis or prognosis;^15^ and (3) the statistical methods were clearly described and the statistical analysis was correct.

**Table 1:** Key items for framing the aim, search strategy, and study inclusion and exclusion criteria for review, in accordance with the PICOTS guidance.

Item	Definition
Population	Patients diagnosed as having KD
Intervention	The model has been used since 2006/01 to 2021/05 to predict IVIG non-response in children with KD, to distinguish children with KD who show IVIG non-response, and to help clinicians to identify such patients early or make long-term treatment plan decisions
Comparator	Alternative models were not considered
Outcomes	Any clinical outcome reported by the IVIG resistance prediction models in children with KD
Timing	Before initiation of treatment with IVIG for children with KD
Setting	Patients diagnosed as showing KD in hospital or medical center/institution

*KD* Kawasaki disease.

### Data extraction and critical appraisal

Two researchers (WS and HH) extracted the data using standardized spreadsheets based on the CHARMS list. From all eligible articles, we extracted information on the first author and year of publication, country, sex and age of the children, study type, study setting, number of predictors in the final model, sample size, model performance metrics, including discrimination (i.e., C-statistic accompanied with 95% CI; predictive values for specificity and sensitivity) and calibration (i.e., slope or plot, and Hosmer–Lemeshow test), and model estimation, including internal and external validation. If important information was missed during the collection process, we contacted the author via email for assistance. Two reviewers (WS, HH) extracted the data, and the third reviewer (SY) analyzed and resolved the conflicts.

PROBAST was used to assess the quality of the included models to identify the ROB that could lead to distortion of the predicted model performance. The evaluator evaluated the ROB for signal problems in the four domains of PROBAST, and the results were categorized as “yes”, “probably yes”, “probably not”, “no”, or “no information”. “Yes” represents a low bias risk, and “no” represents a high bias risk. If the content of relevant signal problems was not provided in the original study, it was judged as indicating that “no information is provided”. The researchers (WS and HH) independently evaluated the bias risk of the included model, and the differences were discussed and negotiated with the consultants (SY and HM) to reach a consensus.

## Results

**Fig. 1:**
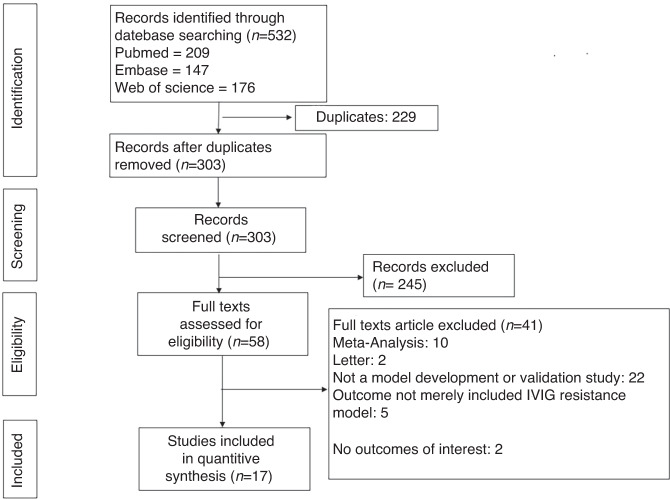
Flow chart of literature selection. Preferred Reporting Items for Systematic Reviews and Meta-analyses (PRISMA) flow diagram of study inclusion for IVIG-resistant Kawasaki disease prediction models.

Figure 1 shows the literature screening process. We identified 532 papers from 3 authoritative databases, of which 229 were duplicates. After screening the title and abstract of the remaining 303 papers, 58 full texts were assessed for eligibility, We further excluded 41 papers because they were meta-analyses (*n* = 10) or letters (*n* = 2), did not involve model development (*n* = 22), did not include the outcome for the IVIG resistance model alone (*n* = 5), or did not include the outcomes of interest (*n* = 2). Finally, a total of 17 studies were included in this study.

### Characteristics of the models

This study summarized the findings of 17 prediction-modeling studies. Most of them were from Asia (China, Japan, Taiwan, and Israel), while two were from Western countries (France, America), and their sample sizes ranged from 105 to 5277.^17,18^ Of the 17 studies, only one used a prospective observational cohort,^19^ while three used prospective methods to test the accuracy of predictive models that were built using retrospective data.^20–22^ Six models were externally validated and only two were validated by the internal population, including a study that involved one internal validation and two external validations.^23^ (Table 2).

**Table 2:** Model characteristics, quality, and validation.

First author (year)	Model characteristics						Model performance		Model estimation
	Country	Sex	Study type	Study setting	Number of predictors (n)	Sample size	Method of discrimination assessed	Method of calibration assessed	Method of validation assessed
Kobayashi T (2006)^21^	Japan	Both	Cohort study	13 medical institutions	7	IVIG Resistance: 148 IVIG Responder: 528	Model AUC: 0.85 (95% CI: 0.81–0.88) external validation AUC: 0.90 (95% CI: 0.85–0.96)	HL and Calibration plot	External validation
Egami K (2006)^25^	Japan	Both	Cohort study	Database of medical institutions	5	IVIG Resistance: 41 IVIG Responder: 279	model AUC: 0.79 (95% CI: 0.73–0.86)	HL	No
Yang S (2019)^23^	China	Both	Cohort study	Multi-center/ hospital	5	IVIG Resistance: 22 IVIG Responder: 90	model AUC: 0.77 (95% CI: 0.72–0.83) internal validation AUC: 0.77 (95% CI: 0.71–0.82) external validation AUC: 0.69 (95% CI: 0.58–0.81) external validation AUC: 0.63 (95% CI: 0.53–0.72)	No	Internal validation two external validation
Wu S (2020)^28^	China	Both	Cohort study	Hospital	5	IVIG Resistance: 31 IVIG Responder: 246	model AUC: 0.750 (95% CI: 0.666–0.834)	No	No
Piram M (2020)^19^	France	Both	Predominantly prospective cohort study	National clinical and biological repository	4	IVIG Resistance: 92 IVIG Responder: 323	Model AUC: 0.725 (sensitivity, 77%; specificity, 60%)	No	No
Wu S (2019)^32^	China	Both	cohort study	Double centers	4	IVIG Resistance: 23 IVIG Responder: 259	Model AUC: 0.891 (95% CI: 0.837–0.945) external validation (sensitivity, 70.0%; specificity, 75.1%)	No	External validation
Fu PP (2013)^33^	China	Both	cohort study	Hospital	5	IVIG Resistance: 211 IVIG Responder: 966	Model AUC: 0.672 (95% CI: 0.631–0.712)	HL	No
Gámez-González LB (2018)^26^	Japan	Both	cohort study	Medical center	5	IVIG Resistance: 101 IVIG Responder: 318	No AUC (sensitivity:76.2%; specificity:64.8%)	No	No
Tan XH (2019)^18^	China	Both	cohort study	hospital	8	IVIG Resistance: 348 IVIG Responder: 4929	AUC: 0.74 (sensitivity:76%; specificity:59%) internal validation AUC: 0.72 (range, 0.65–0.80)	HL	internal validation
Bar-Meir M (2018)^20^	Israel	Both	cohort study	9 medical centers	2	IVIG Resistance: 42 IVIG Responder: 270	AUC: 0.7 (95% CI: 0.6–0.8) external validation AUC: 0.69 (95% CI: 0.59–0.8)	HL	external validation
Wang T (2020)^24^	China	Both	cohort study	hospital	7	IVIG Resistance: 124 IVIG Responder: 520	model AUC: 0.7423 (accuracy: 0.8844; sensitivity: 0.3043; specificity: 0.9919) external validation AUC: not reported	No	external validation
Tang Y (2016)^27^	China	Both	cohort study	hospital	5	IVIG Resistance:46 IVIG Responder: 864	model AUC: 0.77 (95% CI: 0.71–0.82)	HL	No
Hua W (2017)^34^	China	Both	cohort study	hospital	IVIGRKD model:6; IVIGRKD ≤ 6 months old model: 4	IVIG Resistance: 380 IVIG Responder: 1746	model AUC: 0.685 (95% CI: 0.652–0.717) patients ≤ 6 months model AUC: 0.746 (95% CI: 0.665–0.827)	HL	No
Sano T (2016)^35^	Japan	both	retrospective cohort study	seven institutions	3	IVIG Resistance: 22 IVIG Responder: 90	No (sensitivity: 77%, specificity: 86%)	No	No
Tremoulet AH (2008)^30^	America	both	cohort study	2 clinical sites	4	IVIG Resistance: 60 IVIG Responder: 302	No (sensitivity: 73.3%; specificity: 61.9%)	No	No
Lin. M. T (2016)^22^	Taiwan	both	cohort study	hospital	3	IVIG Resistance: 22 IVIG Responder: 159	AUC: 0.86 (95% CI: 0.76–0.97) external validation (sensitivity: 71.4% specificity: 81.0%)	No	external validation
Sato S (2013)^17^	Japan	both	cohort study	hospital	3	IVIG Resistance: 21 IVIG Responder: 84	No (sensitivity: 85.7%; specificity: 77.4%)	No	No

*IVIG* intravenous immunoglobulin.

*H-L* Hosmer–Lemeshow test.

*AUC* area under the curve.

*95% CI* 95% confidence interval.

Logistic regression is the preferred method for building IVIG prediction models (*n* = 15); the literature includes one model that was developed using Lasson regression^18^ and another that was developed using machine learning methods.^24^ Almost all studies (*n* = 15) used univariate analysis to select candidate predictors. Seven studies described a process for handling missing data in this study: one reported that missing values were excluded from the multivariable regression analysis^21^ and one used multiple imputation.^18^ Five studies reported that patients with poorly documented or unclassified clinical data during hospitalization were excluded.^17,25–28^ (Supplementary Table 2).

Discrimination is a model characteristic and is typically assessed with the area under the receiver operating characteristic (AUC).^29^ Thirteen studies used AUC values to assess the discrimination of their models, Four studies did not report performance measurement but demonstrated the sensitivity and specificity of their models.^17,22,26,30^ Calibration was commonly measured with the Hosmer–Lemeshow statistical test.^31^ More than half of the studies (*n* = 10) did not evaluate model fit through calibration methods (Table 2).

### Predictors in the models

Table 3 shows the predictors included in the prediction models of IVIG-resistant Kawasaki disease. All of the predictors included in the model were easily obtained from medical records or laboratory tests. Clinical symptoms included hepatomegaly; polymorphous exanthema; changes around the anus; pre-HR > 146 bpm; pre-BT > 38.8 °C; body weight; rash edema of the extremities; and positive findings for lymphadenopathy. Most predictors were laboratory parameters. Neutrophil-based parameters (including peripheral neutrophil count and neutrophil%) and serum albumin level were the most common predictors (*n* = 8), followed by CRP level (*n* = 5), sodium level (*n* = 5), platelet count (*n* = 5), and total bilirubin level (*n* = 4). Three studies each used the aspartate aminotransferase (AST) level and lymphocyte count; two studies each used the alanine transaminase (ALT) and gamma-glutamyl transferase (GGT) levels and the neutrophil-to-lymphocyte ratio; while the remaining predictors were only used in one model each. Notably, one model found that the inflammatory cytokine (IL-6) level was related to IVIG resistance.^17^

**Table 3:** Predictors included in the 17 prediction models.

First Author (year)	Clinical symptoms	Laboratory Examination	Others
Kobayashi T (2006)^21^		Sodium ≤ 133 mmol/L AST ≥ 100 IU/L Neutrophils ≥ 80% CRP ≥ 10 mg/UL Platelet count ≤ 30.0 × 10^4^/mm^3^	Dates of illness at initial treatment ≤ 4 day Age ≤ 12 months
Egami K (2006)^25^		Platelet count ≤30 × 10^10^/L CRP ≥ 8 mg/dL ALT ≥ 80 IU/L	Infants younger than 6 months Before 4 days of illness
Yang S (2019)^23^		CRP ≥ 90 mg/L Neutrophil ≥ 70% Sodium ion concentration < 135 mmol/L Albumin < 35 g/L Total bilirubin > 20 μmol/L	
Wu S (2020)^28^		NE ≥ 10 × 10^9^/L LY ≤ 3 × 10^9^/L MPV ≥ 10.5 fL ALB ≤ 37 g/L	Age ≤ 24 months
Piram M (2020)^19^	Hepatomegaly	ALT > 30 IU/L Lymphocyte count < 2400/mm^3^	Time to treatment < 5 days
Wu S (2019)^32^		Peripheral NLR ≥ 2.69 MPVLR ≥ 2.78 Serum albumin ≤ 30.7 g/L Serum sodium ≤ 135.2 mmol/L	
Fu PP (2013)^33^	Polymorphous Exanthema; Changes around anus	CRP ≥ 8 mg/dL Neutrophils ≥ 80%	Days of illness at initial treatment
Gámez-González LB(2018)^26^	Pre HR > 146 bpm; Pre BT > 38.8 °C	Neutrophils > 80% Albumin <3.4 g/dL AST > 100 IU/L	
Tan XH (2019)^18^		D-CALs; Na; TBA; P-LYM; PLT RDW, ALB	Age
Bar-Meir M (2018)^20^		Coronary artery abnormalities	Days of illness at presentation <5
Wang T (2021)^24^	Body weight	Platelet count; blood calcium; albumin-to-globulin ratio; total bilirubin; cholesterol	Days of fever prior to hospitalization
Tang Y (2016)^27^	Rash Edema of extremities	Neutrophils ≥ 80% Serum albumin < 35 g/L	Age < 6 months
Hua W (2017)^34^		Sodium ≤ 135 mmol/L NLR ≥ 1.9 Platelet ≤ 350 × 10^9^/L GGT ≥ 25 U/L	Total fever duration ≥ 7 days Delayed diagnosis
Sano T (2006)^35^		CRP ≥ 7.0 mg TB ≥ 0.9 mg AST ≥ 200 IU/L	
Tremoulet AH (2008)^30^		% Bands ≥ 20 GGT ≥ 60 zHgb ≤ −2	Illness day ≤ 4
Lin, M. T (2016)^22^	Positive lymphadenopathy	Albumin < 3.5 g/dL Neutrophil percentage ≥ 60%	
Sato S (2013)^17^		Neutrophil percentage ≥ 75% IL-6 level ≥ 140 pg/mL IL-6 level ≥ 70 pg/mL but ≤ 140 pg/mL	

*CRP* C-reactive protein, *ALT* alanine aminotransferase, *AST* aspartate aminotransferase, *NE* peripheral neutrophil count, *MPV* peripheral mean platelet volume, *LY* peripheral lymphocyte count, *ALB* serum albumin, *NLR* neutrophil-to-lymphocyte ratio, *MPVLR* mean platelet volume-to-lymphocyte ratio, *P-LYM* percentage of lymphocyte, *RDW* red blood cell distribution width, *TBA* total bile acid, *D-CALs* degree of coronary artery lesions, *zHgb* Age-adjusted hemoglobin, *TB* total bilirubin, *GGT* γ-glutamyl transferase.

### Model performance

Table 1 shows the performance metrics for each model. Of the 17 studies, 13 reported AUC values for the development models, ranging from 0.672 (95% confidence interval [CI]: 0.631–0.712)^32^ to 0.891 (95% CI: 0.837–0.945).^33^ The other four models did not report the AUC but reported sensitivity and specificity values. Seven models reported the performance of calibration with the Hosmer–Lemeshow test. Seven models performed external validation (*n* = 5),^19,21,22,24,32^ internal validation (*n* = 1),^18^ or both (*n* = 1).^23^ Six models reported AUC in the external validation and one did not report the AUC but reported the sensitivity and specificity. Two models performed internal validation with AUCs of 0.77 (95% CI: 0.72–0.83)^20^ and 0.72 (95% CI: 0.65–0.80).^23^

### Study quality assessment

**Fig. 2:**
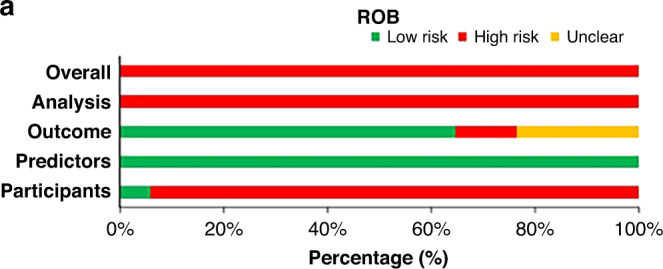
Risk of bias assessment. **a** The risk of bias of the model studies according to the PROBAST.

Table 4 shows the assessment of the ROB of model studies by using PROBAST. Full details are shown in Supplementary Fig. 1. All the models had a high ROB for the analysis domain. Because of the sample size of the training data, predictors were selected on the basis of univariable analysis prior to multivariable modeling; continuous variables were converted to dichotomous variables for inclusion, while predictors were excluded due to missing data or the absence of an explicit mention of the methods for dealing with missing data and inappropriate performance measures. All but one of the articles were retrospective studies, and 16/17 models showed a high ROB for the Participants domain. Six of the 17 articles had predictors that were contained/probably contained predictors in their result definitions that should be considered as high/unclear ROB.^19,22,25,26,32,34^ In contrast, all of the articles showed a low ROB for the Predictors domain. By applying PROBAST, all models were classified as having overall high ROB (Fig. 2).

**Table 4:** Quality assessment for ROB of the included models.

First Author(year)	ROB				Overall
	Participants	Predictors	Outcome	Analysis	ROB
Kobayashi T (2006)^21^	−	+	+	−	−
Egami K (2006)^25^	−	+	−	−	−
Yang S (2019)^23^	−	+	+	−	−
Wu S (2020)^28^	−	+	+	−	−
Piram M (2020)^19^	+	+	−	−	−
Wu S (2019)^32^	−	+	+	−	−
Fu PP (2013)^33^	−	+	?	−	−
Gámez-González LB (2018)^26^	−	+	?	−	−
Tan XH (2019)^18^	−	+	+	−	−
Bar-Meir M (2018)^20^	−	+	+	−	−
Wang T (2021)^24^	−	+	+	−	−
Tang Y (2016)^27^	−	+	+	−	−
Hua W (2017)^34^	−	+	?	−	−
Sano T (2006)^35^	−	+	+	−	−
Tremoulet AH (2008)^30^	−	+	+	−	−
Lin.M.T (2016)^22^	−	+	?	−	−
Sato S (2013)^17^	−	+	+	−	−

*ROB* risk of bias.

+ indicates low ROB;

− indicates high ROB.

## Discussion

This study systematically identified and appraised 17 prediction models published since 2006, and is the first study assessing the ROB of prediction models for IVIG-resistant Kawasaki disease. The prediction models varied widely among different populations; most models were developed in the Asian population, and the predictors used differed across models. Consistent with the results of previous studies, we found that although the models showed some significance in predicting IVIG resistance, clinicians should use them with caution because of their high ROB and limited usefulness in clinically predicting IVIG resistance in children with KD.^35^ The findings of this study also highlighted an urgent need for appropriate sample sizes and validation in large representative populations to ensure that the models could serve as usable tools for population-wide risk assessment. Several recently reported high-risk predictors should be included in these models to improve their predictive performance. In this study, the model quality was often poor, mainly due to limitations in data sources and statistical analyses, and more prospective studies with larger samples as well as standardization of the model development process are required to reduce methodological bias and improve model quality. Some important details of model construction also need to be extended and updated.

### Summary of key findings and further possible directions

#### Model development

The prediction models were developed with a wide variety of populations, with most models established in Asian populations, especially in Japan and China. This finding is consistent with the results suggesting that KD was more prevalent in Asian children.^4^ Sixteen of the 17 models used a retrospective cohort study in which the researchers did not appropriately adjust for the original cohort or registry outcome frequency in the analysis. This may have resulted in a high ROB for the prediction model. However, only one study was based on a prospective cohort design, which may have led to a low ROB.^18,36,37^ Future model development is recommended using prospective studies since data in such studies were collected before the outcome was observed by the researchers themselves, increasing the reliability of the data and generally avoiding recall bias, and tracing the outcomes from cause to effect can shed light on the relationship between exposure factors and disease. Otherwise, for models developed using a case-control study or retrospective cohort study, an inverse sampling fraction can be used to reweight the control and case samples to correct the estimation of baseline risk and obtain corrected absolute predicted probabilities and model calibration measures.^38,39^ Furthermore, all predictors in each study were defined or assessed in a reasonable way, and IVIG resistance was defined consistently, which reduced differences and the potential bias in study results and yielded a low ROB according to PROBAST.

Missing data are commonplace in clinical medical scientific research, irrespective of the study design. Seven of the 17 models dealt with the missing data by directly excluding patients from the incomplete records, which resulted in loss of valuable information and also increased the potential for biased results and biased model performance. In these cases, participants with missing data were more likely to have been automatically removed from the statistical analysis by the statistical package.^15^ Multiple imputation is superior to other methods in terms of bias and accuracy in both development and validation models.^40^ The concept of multiple imputation uses the distribution of observed data to estimate missing data by creating and analyzing multiple data separately and combining them to obtain the overall estimates, variances, and confidence intervals.^41^ Multiple imputation is recommended as the most appropriate method to handle missing data.^42^

Fortunately, all the selected models adopted similar and reliable methods to define and measure the predictors; thus, the predictors adopted uniform definitions, and the blinding method assured measurements, resulting in a low ROB. The predictors were mainly laboratory parameters, clinical symptoms, or digital signs and indicators of the course of the disease, which were easy to obtain and improved the applicability of the models. Nevertheless, the severity of disease, time of starting treatment, and sample size in each model may have led to differences in the final predictors obtained.

The dataset included many features that could serve as candidate predictors. Thirteen models used univariable analysis to select statistically significant (*p* < 0.05) predictors and then used a multivariable prognostic model (logistic regression model) to develop the risk-prediction model.^43^ However, this method does not account for combinations of independent variables even though some predictors that are insignificant in univariable analysis show significance when combined with other predictors. For example, Lin et al.^22^ selected predictors with univariate analysis and found that platelet count was not statistically associated with the IVIG resistance and was not included in the final model. However, platelet counts have been confirmed to predict IVIG-resistant Kawasaki disease.^44^ Selection of predictors based on univariable analysis can only be used for initial screening when the risk factors were numerous. Future models should be encouraged to include candidate predictors based on clinical experience and the literature as well as other well-established predictors. Moreover, some mature predictors and clinical experience should be retained for this purpose.^45^ Furthermore, methods such as Lasson regression and ridge regression can shrink the regression coefficients and are not based on prior statistical tests between predictors and outcomes; these methods are used to alleviate the problem of model overfitting by moving poorly calibrated predicted risks toward the average risk, especially when the model is applied in new patients and few events.^46–48^

More than 85% of the models (15/17) converted a continuous variable after dichotomization, which should be avoided because it can lead to loss of information and biased estimates, even though this approach is helpful for distinguishing clinical results and for disease interpretation.^49^ In such models, patients with similar predicted values slightly above and below the cutoff point were assigned different levels of risk, even though their values were only slightly different. Thus, such models are unstable and have a high ROB if significant nonlinear relationships are present between continuous variables and results in small datasets. We recommend using an approach that can keep predictors continuous.^50,51^ Thus, for classification in studies, the predictor can be classified using a widely accepted predefined cutoff point or adjusted by applying internal and shrinkage techniques. One study used a nomogram to maintain the continuity of predictors.^18^ This strategy does not require the conversion of continuous variables into dichotomous variables, and multiple probability scales can be combined based on the total score to include multiple points of interest in a single chart to inform clinician decision-making.^52^

#### Model evaluation

More than half of the studies evaluated in this study reported discriminative performance, with AUCs ranging from 0.672 to 0.891. Thus, the models had the ability to identify IVIG-resistant Kawasaki disease. However, only seven of the studies reported model calibration performance by Hosmer–Lemeshow test, and five studies reported that calibration or discrimination led to an “N” of 4.7 for PROBAST. To fully evaluate the predictive performance of the model, reviewers must assess both model discrimination and calibration to provide accurate individual probabilities and also focus on the adequacy of the methods used to assess the model.^53^ The most widely reported measures of discrimination were the area under the receiver operating characteristic curve (AUC) as well as the C-statistic, which should be introduced to report the model predictive performance.^54^ Calibration assessment using the Hosmer–Lemeshow test cannot indicate the direction or magnitude of error and has low statistical power, in comparison with calibration plots that better reflect the degree of agreement between the model’s predicted risk and the actual risk of occurrence.^55^

When the number of events per variable (EPVs) is too small and continuous variables are converted to dichotomous variables, univariate analysis or automatic forward and backward methods are used to screen variables, and the overfitting problem becomes more serious while developing prediction models.^15^ External and internal validation are important to ensure optimal fitting of the predicted model. To ensure that predictive models are clinically reliable and well-calibrated, internal validation is required in model development studies. However, only two studies reported internal validation in this study.^20,23^ One study divided the total data into two parts by the random splitting method, using 30% of the study population for internal verification of the model, while the other study used K-fold cross-validation. Internal validation is commonly performed by dividing the dataset into two parts, of which a random small sample is used for internal verification of the model and the remaining data are used to develop the model. However, this approach results in overfitting and optimism of the model, especially when the total sample size is small.^56,57^ Moreover, different random splits may yield different results. Thus, it is recommended to use the original sample data for validation by cross-validation and bootstrap methods,^39^ and external validation of the model in the queue is encouraged to ensure the generality of the prediction model and to evaluate its performance in different independent populations. External validation focuses on whether performance in different regions or populations is consistent with the model from the development queue. It also helps to improve the quality of research results, makes predictive models more credible, and provides a better perspective of the performance of existing models in specific contexts. Six of the models covered in this study were submitted to external validation, although there was a high risk of bias during development, which may have enhanced the confidence of the predictive power.^20–24,33^ Another study with an AUC of 0.77 involved two external validations with good promotion and can be used in multiple centers.^23^ Four models can be used with caution because they were externally validated and showed good discrimination.^20,22,24,33^ The fact that some models were assessed as high-risk because they have not been internally and externally validated does not negate their predictive value, and they can also be considered again with caution if they are well differentiated. The study by Tang et al. showed discrimination (area under the ROC curve) above 0.77 (95% CI 0.71–0.82), although it did not involve internal or external validation, and the new scoring system showed better performance than the Kobayashi and Egami scoring systems in KD patients in East China, so it was advocated for use in this region.^27^

### Challenges and implications of IVIG-resistant Kawasaki disease prediction models

Several barriers exist for the incorporation of predictive models based on available clinical manifestations and laboratory examination data into clinical practice. We have summarized the possible challenges as follows: (1) most current prediction models were based on single-center studies and were built using retrospective datasets without prospective data, which may have resulted in a selection bias that caused poor stability of the model, and for children of different ethnicities and regions, the prediction capability of these scoring systems still needs to be tested. Since the prediction scoring systems showed heterogeneity in different races or different regions of the same race, exploration of a more accurate and perfect scoring system in multicenter and larger sample studies is essential. (2) Kawasaki disease is a form of acute vasculitis involving multiple organs. IVIG is a classical drug for the treatment of Kawasaki disease, and the mechanism of occurrence is not clear at present. Thus, there is a lack of specific laboratory tests to predict IVIG resistance. It may be difficult to obtain a prediction model that can meet clinical expectations only by existing nonspecific laboratory indicators and clinical manifestations. (3) IVIG resistance may be related to genetic factors. Epigenetic inheritance and gene polymorphism may affect the occurrence of IVIG resistance by analyzing the blood samples of children with Kawasaki disease.^58–60^ Therefore, it is necessary to conduct a multicenter genetic study on a large sample of children with IVIG-resistant Kawasaki disease to explore the predictive role of genetic factors on IVIG resistance. (4) Inflammatory factors are dynamic and vary with the course of the disease, and parameters that predict resistance to IVIG may vary depending on the duration of disease prior to treatment with IVIG. However, since laboratory samples were not collected on the same time after the fever, this may have created a bias.^61^ (5) Differences in the production processes of IVIG may also be related to drug resistance, since IVIG prepared with β-propiolactone is more likely to lead to resistance in Kawasaki disease children.^62^ Although IVIG production rules are stipulated by relevant laws, the lack of precision in regulations has resulted in differences in the components of different brands, leading to IVIG resistance in children. Therefore, attention should be paid to the effects of IVIG components on Kawasaki disease.

Prediction models facilitate clinical decision-making, and early warning systems are essential. Rigorously developed and robustly validated predictive models can facilitate early identification and prevent cardiovascular complications, and are a prerequisite for individualized treatment of IVIG-resistant Kawasaki disease. Although IVIG resistance prediction models can help clinicians to identify high-risk KD patients early and administer prompt interventions such as “rescue therapy” (IVIG plus prednisolone or IVIG plus infliximab), several improvements to these models require consideration. The emphasis on model development at the expense of model validation and updating is a common practice in clinical research. Future model research should emphasize external validation of the prediction models identified in this study by using appropriate data sets along with the refinement of internal validation to improve model generality. The prediction models should also be updated according to the latest guideline literature. For example, the 2020 Japanese guidelines for Kawasaki disease state that the prevalence of CAL decreases in children with KD treated with IVIG within 5 days, and therefore recommend the application of IVIG early in the course of the disease. Predictors like “Time to treatment < 5 days” are no longer applicable. On the contrary, the predictive performance of these models can be updated by adding predictors. Plasma IL-6 and TNF-α levels are significantly increased in IVIG-resistant children,^63,64^ which can explain the significant hyponatremia and can also serve as a predictor of IVIG-resistant KD. At the same time, NT-proBNP has also been considered as potential biomarker for KD patients resistant to IVIG treatment.^65^ We acknowledge that some predictors obtained from gene detection, such as interleukin (IL)-2RB, IL-24, BMPR1A, or CHUK, may be associated with IVIG resistance, however, such laboratory tests are complex and time-consuming and may not be easily performed in settings with limited resources; thus, they may not be suitable for prediction models, despite their relevance for identifying IVIG resistance. Moreover, clinicians should consider the predictive performance of the models, establish and select appropriate models according to different regions, populations, and ethnic groups, conduct multicenter and prospective studies, and expand the sample size while strengthening internal and external validation.

## Strengths and limitations

To our knowledge, this is the first study of predictive models for IVIG-resistant Kawasaki disease that describes the existing characteristics in detail. Through a comprehensive search and rigorous screening for study inclusion, we performed a robust ROB assessment of each prediction model by using the new risk-prediction model quality assessment tool PROBAST to understand the quality of current IVIG-resistant models, providing a comprehensive framework for existing studies. Simultaneously, by extracting the predictors used in the included studies, our results yielded a group of candidate predictors that are recommended for future modeling research. The addition of a machine learning model represents a new strategy for future prediction model development. The main limitation of this study was that only English literature was included, and gray literature is not searched. Moreover, gene prediction models were not selected because genetic information was identified as high-risk in PROBAST, and the predictors are not readily available routinely. Nevertheless, we believe that this study can provide clinicians with useful information as well as a reference strategy for future development of predictive models.

## Conclusion

This study summarized and evaluated the findings of 17 studies that reported models for predicting IVIG resistance in Kawasaki disease by assessing their risk of bias (Participants, Predictors, Outcomes, and Analysis). The findings indicated the need to exercise caution since these models carry a high ROB. Our study highlights the need for future model development and validation in accordance with the PROBAST to guide the study design, reduce methodological bias, provide high-quality evidence for clinical practice, continuously improve the predictive performance of the model, and ensure ease of use and generalizability of the model.

## Supplementary information

Supplementary information

## Data Availability

All data relevant to the study are included in the article or uploaded as supplementary information.
