# Supplementary material for: Risk-prediction models for intravenous immunoglobulin resistance in Kawasaki disease: Risk-of-Bias Assessment using PROBAST
Source: Pediatr Res. 2023 Mar 24;94(3):1125–35. doi: 10.1038/s41390-023-02558-6 (PMC10444619; doi:10.1038/s41390-023-02558-6)
Supplement: Supplementary file 1 — Supplementary information [file 41390_2023_2558_MOESM1_ESM.pdf]

Supplementary Table 1. Search strategy in PubMed and Embase

Search strategy in PubMed	
#1	predict[tiab] OR score[tiab] OR nomogram[tiab] OR model[tiab]
#2	kawasaki disease [tiab] OR mucocutaneous lymph node syndrome [tiab]
#3	IVIG resistance [tiab] OR IVIG unresponsiveness [tiab]
#4	#2 AND #3
#5	English[Language]
#6	Journal Article[Publication Type]
#7	2006/01-2021/05[Date - Publication]
#8	#1 AND #4 AND #5 AND #6 AND #7
Search strategy in Embase	
#1	predict[tiab] OR score[tiab] OR nomogram[tiab] OR model[tiab]
#2	kawasaki disease [tiab] OR mucocutaneous lymph node syndrome [tiab]
#3	IVIG resistance [tiab] OR IVIG unresponsiveness [tiab]
#4	#2 AND #3
#5	english:la
#6	article:it AND [2006/01-2021/05]/py
#7	#1 AND #4 AND #5 AND #6

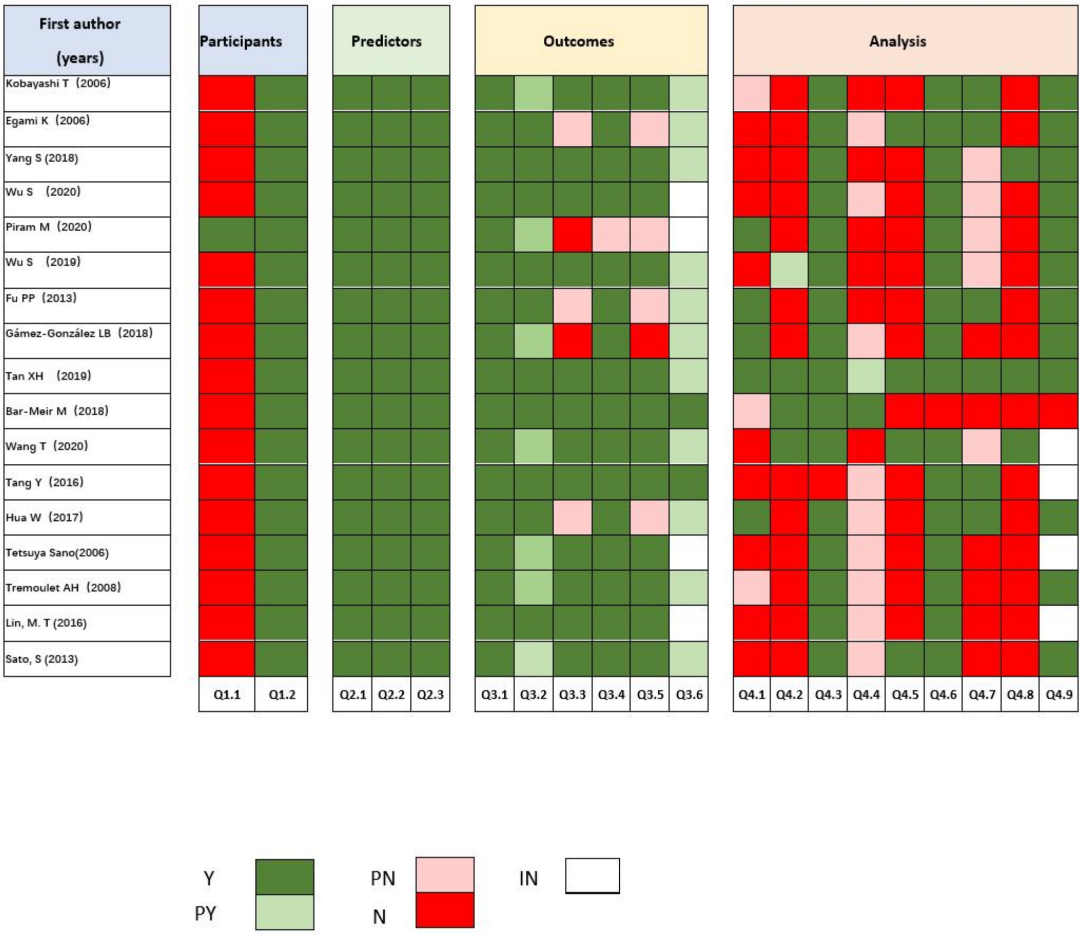

Supplementary Figure 1.

Each signal question was answered as yes (Y, denoted by bottle green), probably yes (PY, denoted by light green), no (N, indicated by deep red), probably no (PN, indicated by light red), or no information (NI, denoted by white). Q = question.

Q1.1: Were appropriate data sources used, e.g., cohort, RCT, or nested case–control study data?
Q1.2: Were all inclusions and exclusions of participants appropriate?
Q2.1: Were predictors defined and assessed in a similar way for all participants?
Q2.2: Were predictor assessments made without knowledge of outcome data?
Q2.3: Are all predictors available at the time the model is intended to be used?
Q3.1: Was the outcome determined appropriately?
Q3.2: Was a prespecified or standard outcome definition used?
Q3.3: Were predictors excluded from the outcome definition?
Q3.4: Was the outcome defined and determined in a similar way for all participants?
Q3.5: Was the outcome determined without knowledge of predictor information?
Q3.6: Was the time interval between predictor assessment and outcome determination appropriate?
Q4.1: Were there a reasonable number of participants with the outcome?
Q4.2: Were continuous and categorical predictors handled appropriately?
Q4.3: Were all enrolled participants included in the analysis?
Q4.4: Were participants with missing data handled appropriately?
Q4.5: Was selection of predictors based on univariable analysis avoided? [development studies only]
Q4.6: Were complexities in the data (e.g., censoring, competing risks, sampling of control participants) accounted for appropriately?
Q4.7: Were relevant model performance measures evaluated appropriately?
Q4.8: Were model overfitting and optimism in model performance accounted for? [development studies only]
Q4.9: Do predictors and their assigned weights in the final model correspond to the results from the reported multivariable analysis? [development studies only]

Supplementary table 2. Summary of methods used in the included models

First author (year)	Model development				
	**Selection of candidate predictors**	**Regression method**	**Treatment of continuous variables**	**Missing data of predictors**	**Methods for handling missing data for model development**
**Kobayashi T（2006）[21]**	demographic variables (age in months, sex, and days of illness at initial treatment) plus laboratory variables that had been selected by univariable analysis	Logistic	continuous predictors are converted into ≥2 categories	Yes	Patients with missing values were excluded from the multivariable regression analysis
**Egami K（2006）[25]**	predictive variables were selected by univariable analysis.	Logistic	continuous predictors are converted into ≥2 categories	Not report	Not report
**Yang S（2019）[23]**	predictive variables were selected by univariable analysis	Logistic	continuous predictors are converted into ≥2 categories	Yes	104 patients with missing values were excluded
**Wu S（2020）[28]**	predictive variables were selected by univariable analysis and together with the demographic characteristics (age, sex)	Logistic	continuous predictors are converted into ≥2 categories	Not report	Not report
Piram M（2020）[19]	predictive variables were selected by univariable analysis	Logistic	continuous predictors are converted into ≥2 categories	Yes	Excluding the missing information
**Wu S（2019）[32]**	predictive variables were selected by univariable analysis	Logistic	continuous predictors are converted into ≥2 categories，and adjusted by the previous classical literature and clinical practice	Yes	20 patients with missing values were excluded
**Fu PP（2013）[33]**	predictive variables were selected by univariable analysis	Logistic	continuous predictors are converted into ≥2 categories	Yes	58 children had incomplete clinical or laboratory data
**Gámez-González LB（2018）[26]**	predictive variables were selected by univariable analysis	Logistic	continuous predictors are converted into ≥2 categories	Not report	Not report
**Tan XH （2019）[18]**	predictive variables were selected by univariable analysis, least absolute shrinkage and selection operator (LASSO)	LASSO	use nomogram to maintain continuity of variables	Yes	For the variables with miss rate <25%, multiple imputation was used， the unconjugated bilirubin was excluded due to its missing rate of 58%.
**Bar-Meir M（2018）[20]**	predictive variables were selected by univariable analysis	Logistic	continuous variables are defined in the same way	Yes	Not report
**Wang T（2020）[24]**	risk factors drawn from previous studies or literature	Machine learning algorithms	maintain continuity of variables	Yes	Excluding the missing information
**Tang Y（2016）[27]**	predictive variables were selected by univariable analysis	Logistic	continuous predictors are converted into ≥2 categories	Not report	Not report
**Hua W（2017）[34]**	predictive variables were selected by univariable analysis	Logistic	continuous predictors are converted into ≥2 categories	Yes	Not report
**SaNo T（2017）[35]**	predictive variables were selected by univariable analysis	Logistic	continuous predictors are converted into ≥2 categories	Yes	Not report
**Tremoulet AH（2008）[30]**	predictive variables were selected by univariable analysis	Logistic	continuous variables were converted to dichotomous variables	Yes	Not report
**Lin.M. T (2016)[22]**	predictive variables were selected by univariable analysis	Logistic	continuous variables were converted to dichotomous variables	Yes	Not report
**Sato, S (2013)[17]**	predictive variables were selected by univariable analysis	Logistic	continuous variables were converted to dichotomous variables	Not report	Not report
